# Expression of Foxp3 in Colorectal Cancer but Not in Treg Cells Correlates with Disease Progression in Patients with Colorectal Cancer

**DOI:** 10.1371/journal.pone.0053630

**Published:** 2013-01-30

**Authors:** Mia Kim, Tanja Grimmig, Martin Grimm, Maria Lazariotou, Eva Meier, Andreas Rosenwald, Igor Tsaur, Roman Blaheta, Uwe Heemann, Christoph-Thomas Germer, Ana Maria Waaga-Gasser, Martin Gasser

**Affiliations:** 1 Department of Surgery I, University of Wuerzburg, Wuerzburg, Germany; 2 Department of Surgery I, Molecular Oncology and Immunology, University of Wuerzburg, Wuerzburg, Germany; 3 Department of Oral and Maxillofacial Plastic Surgery, University of Tuebingen, Tuebingen, Germany; 4 Institute of Pathology, University of Wuerzburg, Wuerzburg, Germany; 5 Department of Urology, University of Frankfurt, Frankfurt, Germany; 6 Department of Nephrology, University of Munich, Klinikum rechts der Isar, Munich, Germany; Sun Yat-sen University Medical School, China

## Abstract

**Background:**

Regulatory T cells (Treg) expressing the transcription factor forkhead-box protein P3 (Foxp3) have been identified to counteract anti-tumor immune responses during tumor progression. Besides, Foxp3 presentation by cancer cells itself may also allow them to evade from effector T-cell responses, resulting in a survival benefit of the tumor. For colorectal cancer (CRC) the clinical relevance of Foxp3 has not been evaluated in detail. Therefore the aim of this study was to study its impact in colorectal cancer (CRC).

**Methods and Findings:**

Gene and protein analysis of tumor tissues from patients with CRC was performed to quantify the expression of Foxp3 in tumor infiltrating Treg and colon cancer cells. The results were correlated with clinicopathological parameters and patients overall survival. Serial morphological analysis demonstrated Foxp3 to be expressed in cancer cells. High Foxp3 expression of the cancer cells was associated with poor prognosis compared to patients with low Foxp3 expression. In contrast, low and high Foxp3 level in tumor infiltrating Treg cells demonstrated no significant differences in overall patient survival.

**Conclusions:**

Our findings strongly suggest that Foxp3 expression mediated by cancer cells rather than by Treg cells contribute to disease progression.

## Introduction

The identification of CD4+CD25+ T regulatory cells (Treg) has been shown to play a crucial role in maintaining immunologic tolerance. The transcription factor forkhead box protein P3 (Foxp3) has been identified as a key player in Treg function and is an obligate marker of CD4+CD25+ Treg [1]. Some subclasses of Treg exert their suppressive influence via the expression of immunosuppressive cytokines, such as interleukin (IL)-10 and transforming growth factor (TGF)-β [2], [3]. A high density of tumor infiltrating Foxp3+ Treg in tumor specimen has been associated with poor outcome in various solid tumors, including ovarian [4], pancreatic [5], and hepatocellular carcinoma [6]. These findings suggest a crucial role for Treg in different tumor entities. Thus targeting Treg may have an important impact on immunotherapeutic anti-cancer strategies and the clinical outcome of cancer patients [7].

Treg are suspected of reducing T cell activity but it is not known whether the presence of Treg may have an impact on the clinical course and on tumor related survival of patients with CRC. The prognostic significance of Treg detection in patients with limited and advanced disease remains still controversial. To date, few studies have analyzed infiltrating Treg in CRC using Foxp3+ staining. A recent study demonstrated that Treg density was higher in locally limited than in metastatic disease but was not associated with the survival of CRC patients [8]. Contrary to the findings observed in most other human carcinomas, no significant relation between the absolute number of Foxp3+ infiltrating T cells and prognosis was observed in several studies with CRC patients. Furthermore, some other studies suggest that a high frequency of tumor infiltrating Foxp3+ Treg is associated with favourable prognosis in CRC [9].

More recent clinical data from lung [10], breast [11], [12], pancreatic [13], hepatocellular [14], and urinary bladder cancer [15] as well as melanoma [16] provided first evidence for a Foxp3 expression also in tumor cells. However, the biological significance of Foxp3 expression in cancer cells of patients with CRC remains unknown. In particular, the contribution of Foxp3 expression related to tumor cells as compared to the expression related to Treg in clinical CRC has not been evaluated so far. Therefore, the purpose of this study was to evaluate Foxp3 expression between tumor infiltrating Treg and cancer cells in patients with CRC at different stages of the disease as well as to discriminate its prognostic significance over the long-term.

## Results

### Detection of CD4, CD25, Foxp3 and immunosuppressive cytokines IL-10 and TGF-β genes by RT-qPCR and immunohistochemical analysis

**Figure 1:**
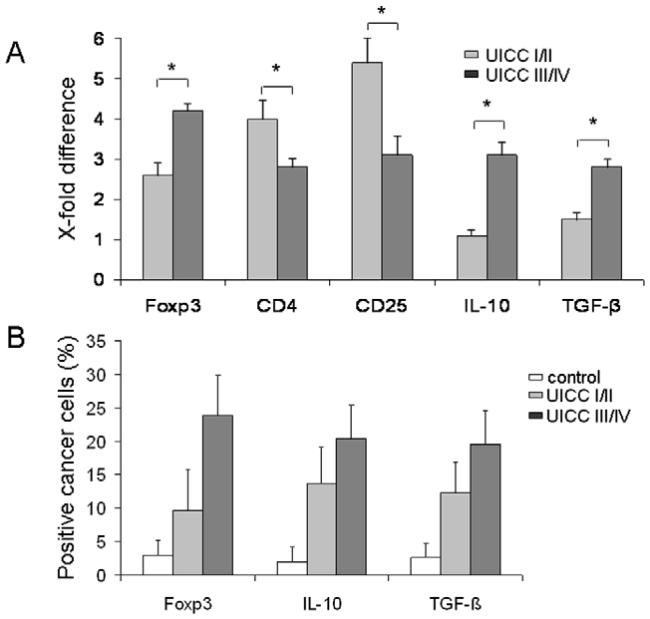
Gene and protein expression analysis of CD4, CD25, Foxp3, IL-10, and TGF-β from patients with CRC (n = 65) by RT-qPCR and immunohistochemical analysis of cancer cells in early (UICC I/II) and late stage (UICC III/IV) of the disease. (**A**) Significantly increased gene expression of CD4 and CD25 at stage UICC I/II compared to tumors at stage UICC III/IV. Gene expression of Foxp3, IL-10, and TGF-β was significantly decreased at stage I/II as compared with those at UICC III/IV. The normalization was performed with normal tissue. The relative quantification value, fold difference, is expressed as 2^−ΔΔCt^. *p<0.001. (**B**) Foxp3+, IL-10+, and TGF-β+ expressing cancer cells increased from UICC I/II to UICC III/IV compared to normal tissue. The result of the staining was expressed in percentages (%) positivity. All values were expressed as mean ± SD; all pairwise tests (Tukey) result in p<0.001 with exception of control vs. UICC I/II in Foxp3^+^ (p<0.050).

To analyze whether CD4, CD25, Foxp3, IL-10, and TGF-β expression in CRC may be associated with clinical tumor progression we investigated tumors of limited disease (UICC I/II) and advanced disease (UICC III/IV). RT-qPCR analysis showed significantly increased gene expression of CD4 and CD25 in limited disease tumors (UICC I/II) compared to tumors of advanced disease (UICC III/IV). In accordance to this finding, gene expression of Foxp3 and immunosuppressive cytokines IL-10 and TGF-β was significantly decreased in limited disease tumors (UICC I/II) compared to those of advanced disease (UICC III/IV) (***Figure*** **** ***1A***).

Next, we examined the expression of Foxp3 and immunosuppressive cytokines IL-10 and TGF-β in cancer cells. As shown in ***Figure*** **** ***1B***, Foxp3+, IL-10+, and TGF-β+ expressing cancer cells increased from early to late stages of disease compared to normal tissue. Foxp3^+^ expressing cancer cells were found in 60 out of 65 tumor cases (n = 60/65, 92.3%). Additionally, we stained 36 of the overall 65 cases with a different anti-Foxp3 antibody (clone 2481) and confirmed the results (data not shown).

### Immunohistochemical analysis of CD4+, CD25+, Foxp3+, and immunosuppressive cytokines IL-10+ and TGF-β+ in Treg

**Figure 2:**
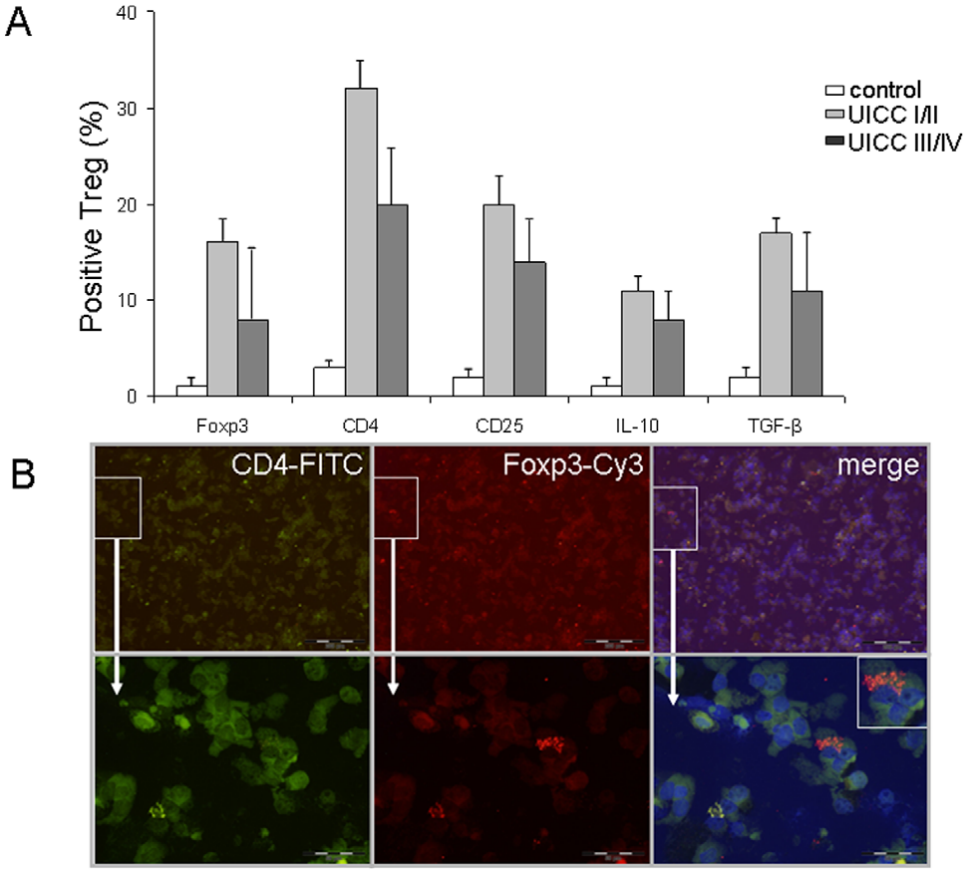
Immunohistochemical analysis of CD4+, CD25+, Foxp3+, IL-10+, and TGF-β+ expression in Treg from patients with CRC (n = 65) in early (UICC I/II) and late stage (UICC III/IV) of the disease. (**A**) Increased CD4+, CD25+, Foxp3+, IL-10+, and TGF-β+ expression at stage UICC I/II as compared with those at UICC III/IV. The result of the staining was expressed in percentages (%) positivity. All values were expressed as mean ± SD. All pairwise tests result in p<0.001 with three exceptions: Foxp3^+^, control vs. UICC III/IV, p = 0.091; IL-10^+^, UICC I/II vs. UICC III/IV, p = 0.021; TGF-ß^+^, UICC I/II vs. UICC III/IV, p = 0.020. (**B**) Representative example of an immunofluorescence double staining of Foxp3+ and CD4+ in Treg. Foxp3 expression was mainly observed on CD4+ Treg (arrow) (×400 magnification). FITC, green Fluoresceinisothiocyanate, Cy3, indocarbocyanin red, and DAPI 4′,6-Diamidino-2- phenylindoldihydrochlorid blue – nuclear counterstaining.

We next examined Treg and cancer cells for a detailed expression analysis of Foxp3, IL-10, and TGF-β by immunohistochemistry. First, we examined the expression of CD4+, CD25+, Foxp3+, and immunosuppressive cytokines IL-10 and TGF-β in Treg. As shown in ***Figure*** **** ***2A***, increased CD4+, CD25+, Foxp3+, IL-10+, and TGF-β+ expression was observed in limited disease tumors (UICC I/II) as compared to advanced disease tumors (UICC III/IV) and normal tissue. Overall, Foxp3+ expressing Treg in different amounts were found in 61 out of 65 tumors of the patients (n = 61/65, 93.8%). Immunofluorescence double staining demonstrated that Foxp3+ Treg were of a CD4+ T cell phenotype (***Figure*** **** ***2B***). Only a very small portion of less than 5% of cells was found to represent a CD8+ T cell subpopulation expressing Foxp3 (data *not shown*).

### Gene and protein analysis of Foxp3 expression in cancer cells of patients with CRC and in human colon cancer cell lines by RT-qPCR, immunofluorescence double staining and flow cytometry

**Figure 3:**
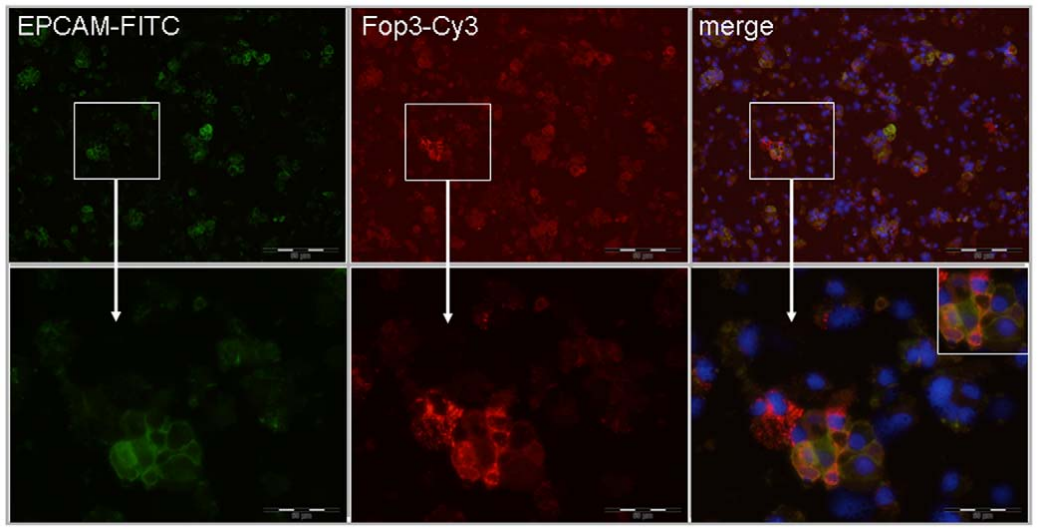
Immunofluorescence double staining of Foxp3 and EPCAM in cancer cells from patients with CRC. Representative example of an immunofluorescence double staining, showing Foxp3 expression and EPCAM costaining in cancer cells of patients with CRC (×100 magnification above; ×400 magnification below). FITC, green Fluoresceinisothiocyanate, Cy3, indocarbocyanin red and DAPI 4′,6-Diamidino-2- phenylindoldihydrochlorid blue – nuclear counterstaining.

**Figure 4:**
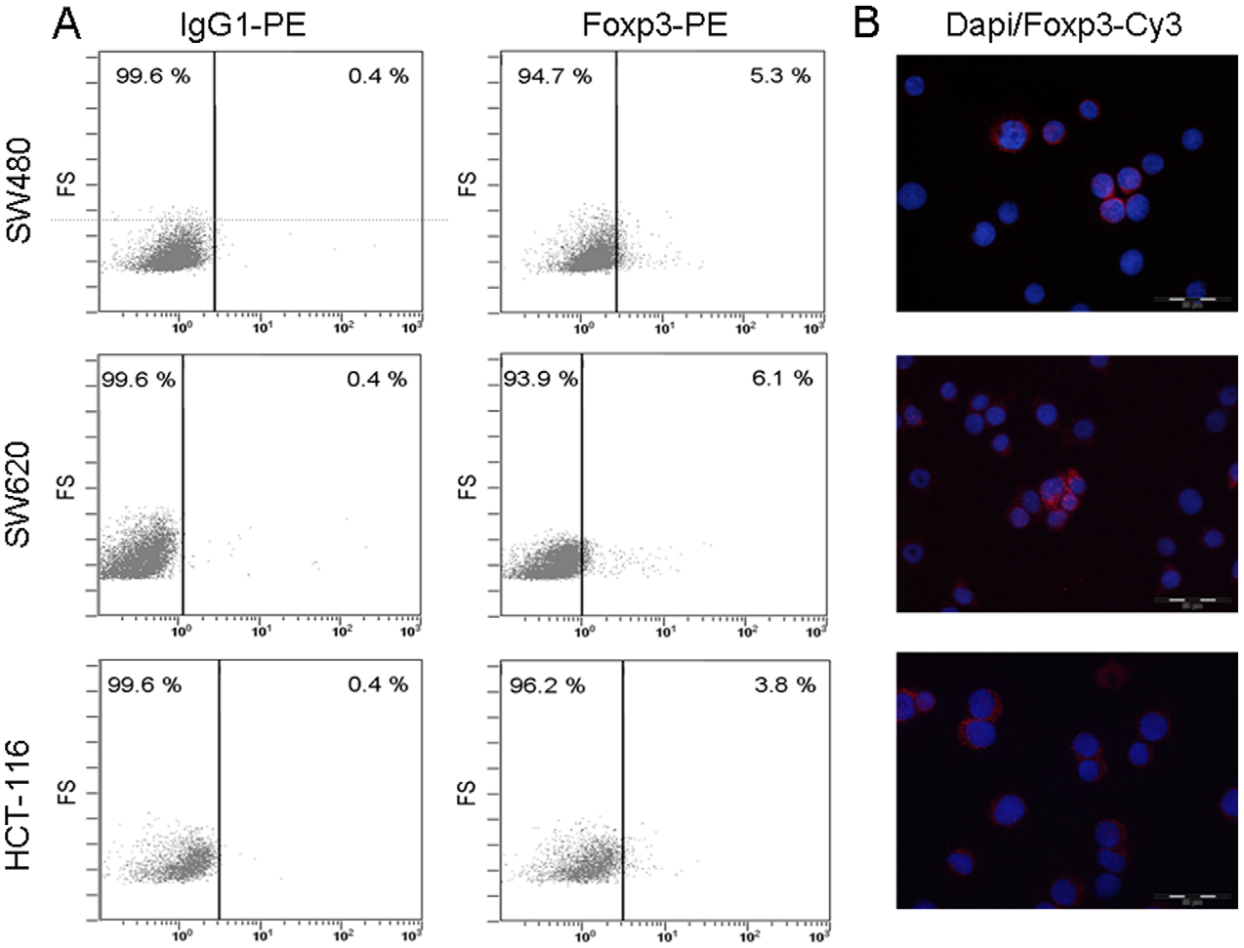
Protein expression of Foxp3 in colon cancer cell lines by flow cytometry and immunofluorecence double staining analysis. (**A**) Flow cytometry assay of Foxp3 expression in SW480, SW620, and HCT-116 colon cancer cell lines compared to isotype control. 3.8% to 6.1% of colon cancer cells express Foxp3; PE: phycoerythrin; FS: forward scatter linear. (**B**) Representative examples of immunofluorescence double staining of Foxp3+ expression in SW480, SW620, and HCT-116 cancer cells. Cy3, indocarbocyanin red and DAPI 4′,6-Diamidino-2- phenylindoldihydrochlorid blue – nuclear counterstaining (×400 magnification).

First we demonstrated Foxp3 expression in cancer cells of patients with CRC using immunofluorescence double staining (***Figure*** **** ***3***). To confirm the Foxp3 expression in tumor cells we performed RT-qPCR, FACS analysis and immunofluorescence double staining analyses in different human colon cancer cell lines (SW480, SW620, and HCT-116). As demonstrated by FACS 3.8% to 6.1% of the cancer cells indeed expressed Foxp3 (***Figure*** **** ***4A and B***). *G*ene analysis by RT-qPCR confirmed its expression (relative expression in the cell lines ranged between 1.76 and 2.08, ***Table*** **** ***1***).

**Table 1:** Quantitative Real Time PCR analysis of Foxp3 expression in colon cancer cell lines.

	ΔCt	Relative expression
**SW480**	15.15	1.76
**SW620**	20.54	2.08
**HCT-116**	19.09	1.95

Foxp3 is expressed in the human colon cancer cell lines SW480, SW620, and HCT-116 (n = 5).

### Correlation of Foxp3+ cancer cells with the expression of immunosuppressive cytokines IL-10 and TGF-β

**Figure 5:**
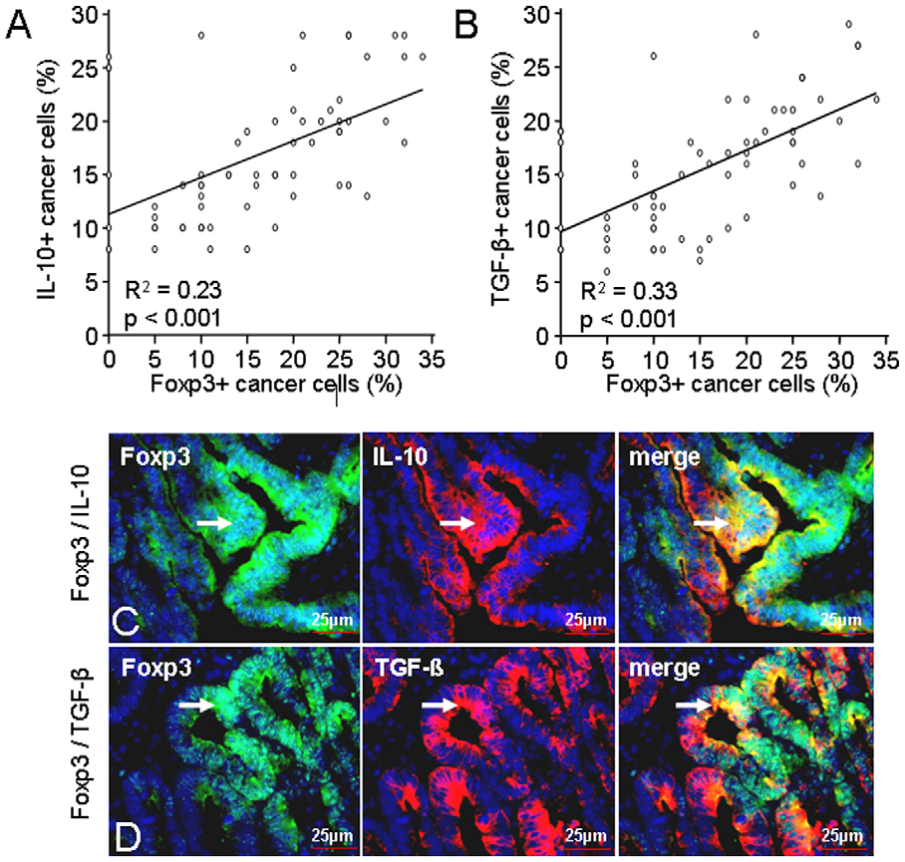
Correlation of Foxp3+ cancer cell expression with immunosuppressive cytokines IL-10+ and TGF-β+ and immunofluorescence double staining. (**A/B**) Significant correlation of Foxp3+ cancer cell expression with the expression of IL-10 (**A**) and TGF-β (**B**). Regression analysis; R^2^, coefficient of determination. (**C/D**) Representative example of an immunofluorescence double staining of IL-10+ (**C**) and TGF-β+ (**D**) in Foxp3+ cancer cells (arrows). FITC green Fluoresceinisothiocyanate, Cy3 red and DAPI 4′,6-Diamidino-2- phenylindoldihydrochlorid blue – nuclear counterstaining.

To examine whether the expression of the immunosuppressive cytokines IL-10 and TGF-β corresponded with the Foxp3+ expressing cancer cells we stratified in two different groups according to the percentages of expression in the immunohistochemical analysis. Considering Foxp3+ expression in cancer cells as a continuous variable, regression analysis showed that Foxp3+ cancer cell expression had a weak but significant direct correlation with the expression of the immunosuppressive cytokines IL-10 (R^2^ = 0.23, p<0.001, n = 65; r = 0.48) and TGF-β (R^2^ = 0.33, p<0.001, n = 65; r = 0.57) (***Figure*** **** ***5A and B***).

Immunofluorescence double staining indicated the expression of the immunosuppressive cytokines IL-10 and TGF-β in Foxp3+ expressing cancer cells (arrows) (***Figure*** **** ***5C and D***).

### Correlation of Foxp3+ Treg with Foxp3+ cancer cells

**Figure 6:**
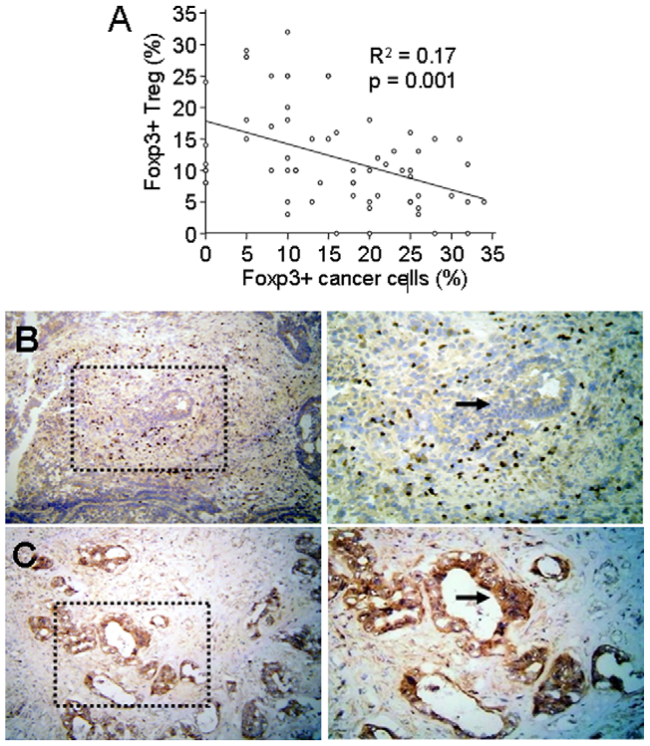
Correlation of Foxp3+ Treg with Foxp3+ cancer cell expression and immunohistochemical analysis of Foxp3+ Treg and Foxp3+ cancer cells. (**A**) Significant inverse correlation of Foxp3+ cancer cell expression with the Foxp3 Treg expression. Regression analysis; R^2^, coefficient of determination. (**B**) Increased numbers of Foxp3+ Treg in Foxp3 negative cancer tissue (arrow). (**C**) Only occasionally or no Foxp3+ Treg in Foxp3 positive cancer tissue (arrow). DAB brown color, Haemalaun blue color – nuclear counterstaining. Representative example demonstrates area of magnifications x100 (left) and x200 (right).

To examine whether Foxp3+ Treg expression corresponded with the Foxp3+ cancer cell expression, we stratified two different groups according to percentages expression of immunohistochemical analysis. Considering the Foxp3+ cancer cell expression as a continuous variable, regression analysis showed that Foxp3+ cancer cell expression had a weak but significant inverse correlation with the Foxp3+ Treg expression (R^2^ = 0.17, p = 0.01, n = 65; r = −0.41) (***Figure*** **** ***6A***). Immunohistochemistry showed increased Foxp3+ Treg expression in Foxp3 negative cancer stromal tissue (arrow) (***Figure*** **** ***6B***). In contrast, there was no or negligible Foxp3+ Treg expression found in Foxp3 positive cancer tissue (arrow) (***Figure*** **** ***6C***).

### Overall survival

Multivariate Cox regression analysis was performed stepwise including age, gender, primary tumor (colon or rectum), UICC (I/II or III/IV), depth of tumor invasion (T category 1/2 or 3/4), differentiation (1/2 or 3/4), lymph node metastasis (N category), Foxp3 (%), Treg (%), TGF-ß (%), and IL-10 (%). The stepwise procedure kept in the model the N category and Foxp3 expression in colon cancer cells as prognostic parameters (Chi-quadrat statistics, p<0.01, ***Table*** **** ***2***).

**Table 2:** Multivariate analysis of prognostic factors of the study population.

	Unfavorable factor	Hazard ratio (HR)	95% CI of HR	?^2^ p-values
**Lymph nodes metastasis**	Positive	8.97	2.28 to 35.31	0.002
**Foxp3+ cancer cells**	High	1.09	1.02 to 1.14	0.006

Lymph node metastasis (N category) and Foxp3+ were found to be prognostic parameters of survival (p<0.01, Chi-Quadrat).

### Univariate results using Kaplan-Meier

**Figure 7:**
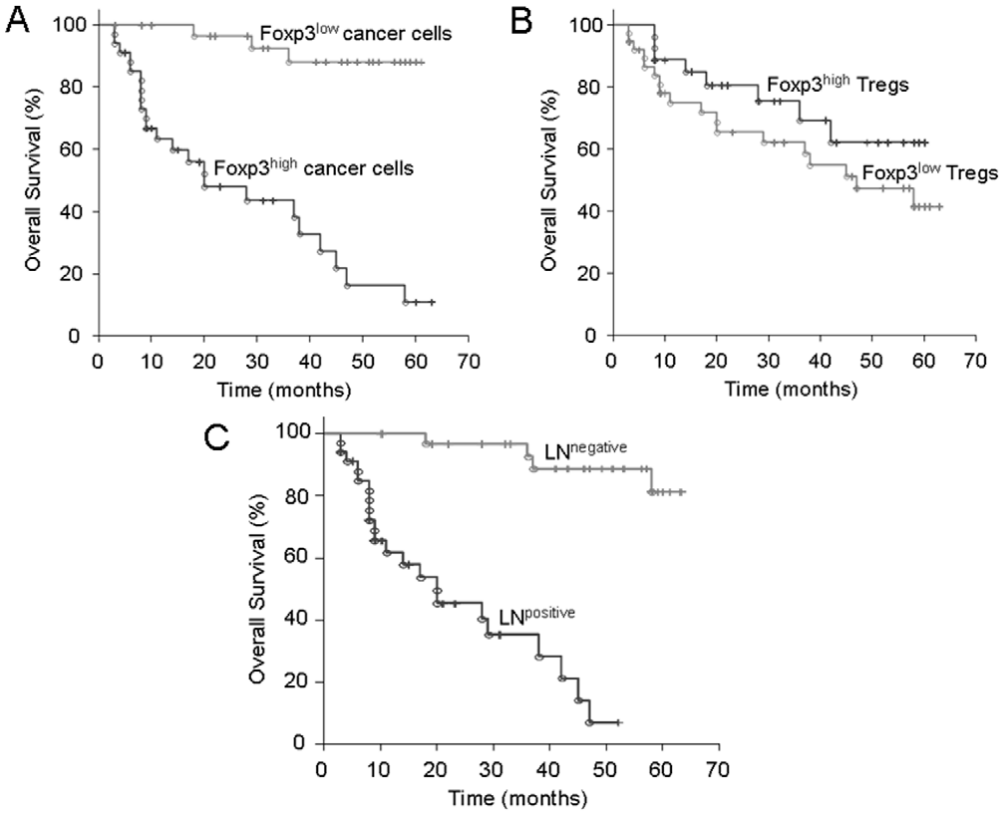
Overall survival of cancer patients with Foxp3+ cancer cell expression in their CRCs compared with the overall survival of those with infiltrated Foxp3+ Treg in their tumors. (**A**) Patients with high Foxp3+ cancer cell expression (>16%, as mean cut-off) had a poorer prognosis than those with low Foxp3+ cancer cell expression profiles (<16%; mean cut-off: 16%), (p<0.001, Log-Rank test). (**B**) No significant difference in the overall survival comparing patients with low and high Foxp3+ Treg expression profiles (mean cut-off: 12%), (p = 0.202, Log-Rank test). (**C**) Patients with lymph node metastasis had a poorer prognosis than those without lymph node metastasis (p<0.001, Log-Rank test). The times of the censored data are indicated by short vertical lines.

The identified prognostic factors from Cox regression model are presented in Figures 7A and C. The mean value of Foxp3+ cancer cell expression by immunohistochemical analysis for all studied tissue samples of the 65 tumors was determined at 16%. Among patients with CRC, those with high Foxp3+ cancer cell expression (>16%) had a poorer prognosis than those with low Foxp3+ expression levels (<16%) (p<0.001, Log-Rank test) (***Figure*** **** ***7A*** ***and*** ***Table*** **** ***3***).

**Table 3:** Clinicopathological characteristics of the study population and discrimination of Foxp3 expression profiles.

		Total	Foxp3 (cancer cells)			Foxp3 (Treg)		
			Low	High	Test	Low	High	Test
**Cases (n)**		65 (100%)	31 (100%)	34 (100%)		38 (100%)	27 (100%)	
**Age (y; mean ± sd)**		64.0±5.8	63.9±5.7	64.2±5.9	n.s.	64.2±6.0	63.8±5.6	n.s.
**Gender**								
	Male	37 (57%)	18 (58%)	19 (56%)	n.s.	21 (55%)	16 (59%)	n.s.
	Female	28 (43%)	13 (42%)	15 (44%)		17 (45%)	11 (41%)	
**Primary tumor**								
	Colon	26 (40%)	12 (39%)	14 (41%)	n.s.	16 (42%)	10 (37%)	n.s.
	Rectum	39 (60%)	19 (61%)	20 (59%)		22 (58%)	17 (63%)	
**Differentiation**								
	G1	12 (18%)	7 (22%)	5 (15%)	n.s.	4 (11%)	8 (30%)	n.s.
	G2	31 (48%)	16 (52%)	15 (44%)		21 (55%)	10 (37%)	
	G3/4	22 (34%)	8 (26%)	14 (41%)		13 (34%)	9 (33%)	
**Depth of invasion**								
	pT1	14 (22%)	10 (32%)	4 (12%)	<0.001	8 (21%)	6 (22%)	n.s.
	pT2	23 (35%)	14 (45%)	9 (27%)		13 (34%)	10 (37%)	
	pT3	17 (26%)	7 (23%)	10 (29%)		10 (26%)	7 (26%)	
	pT4	11 (17%)	-	11 (32%)		7 (19%)	4 (15%)	
**Lymph node metastasis**								
	pN0	31 (48%)	25 (81%)	6 (18%)	<0.001	14 (37%)	17 (63%)	0.038
	pN1–3	34 (52%)	6 (19%)	28 (82%)		24 (63%)	10 (37%)	
**UICC stage**								
	UICC I	15 (23%)	13 (42%)	2 (6%)	<0.001	8 (21%)	7 (26%)	n.s.
	UICC II	19 (29%)	16 (52%)	3 (9%)		7 (18%)	12 (45%)	
	UICC III	22 (34%)	1 (3%)	21(62%)		16 (43%)	6 (22%)	
	UICC IV	9 (14%)	1 (3%)	8 (23%)		7 (18%)	2 (7%)	
**Mean OS (m)**			35	27		38	35	
**Median OS (m)**			n.a.	20		47	n.a.	
**Log-Rank test**			p<0.001			p = 0.204		

Patients with CRC and with high Foxp3+ cancer cell expression (≥16%; mean cut-off 16%) had a poorer prognosis than those with low Foxp3+ cancer cell expression (<16%). No significant difference in the overall survival comparing patients with low and high Foxp3+ Treg expression (mean cut-off: 12%). Y, years; G, grading; UICC, International Union against Cancer; R, residual tumor; OS, overall survival; m, months; n.s., not significant; n.a., not applicable.

Considering immunohistochemical analysis of the samples from the 65 tissue samples for Foxp3+ Treg expression the mean value was calculated at 12%. There was no significant difference in the overall survival comparing patients with low and high Foxp3+ Treg expression levels (>12% or <12%) (p = 0.204, Log-Rank test) (***Figure*** **** ***7B*** ***and*** ***Table*** **** ***3***).

In patients without lymph node metastasis were significant differences in overall survival compared to patients with lymph node metastasis (p<0.001, Log-Rank test) (***Figure*** **** ***7C***).

Other parameters such as TGF-ß, IL-10, UICC, and T category showed additionally significant differences in overall survival for the corresponding lower expression and grading, respectively (p<0.001, Log-Rank tests).

Age, gender, primary tumor, and histological differentiation were not associated with prognosis in univariate analysis.

## Discussion

The current study provides for the first time evidence of a significantly increased tumor-related expression of the transcription factor Foxp3 in colorectal cancer cells that is associated with adverse prognosis. Detailed protein and gene analysis was used to study its expression profiles in each tumor tissue of the patients. Based on more recently described clinical findings of a Foxp3 expression in cancer cells of tumors like lung, hepatocellular, and urinary bladder cancer as well as melanoma we suggested that elevated Foxp3 expression levels were not necessarily associated to Treg alone but also to cancer cells [10], [14], [15]. Expression of Foxp3 by cancer cells would enable them to downregulate effector T cell responses directed against the tumor. This would give clinical evidence for an effective mechanism of a direct tumor-derived evasion from immunological destruction in CRC. By discriminating Foxp3 expression of cancer cells from infiltrating Treg and correlating with overall survival over a long-term follow-up we provide new insights in its prognostic significance.

In accordance with previous studies in various malignant diseases we found significantly higher numbers of infiltrating Treg (CD4+CD25+Foxp3+) in all CRC samples compared with normal colon tissues [4]–[6], [17]–[20]. However, the association of Foxp3 expression in Treg and its impact on overall survival remains controversial. Ambiguous data suggest that tumor infiltration by Foxp3+ Treg is not always associated with a poor prognosis, but, on the contrary, can be associated with an improved prognosis in some cancer types like in CRC [8], [9], [21], [22]. Moreover, no significant relation was observed between the absolute number of Foxp3+ Treg and prognosis in CRC [23], [24]. Improved survival and the potentially protective role of Treg might be explained by their capacity of reducing the development of an aggressive and cytotoxic, potentially proliferogenic cytokine milieu, which is the basis for an inflammation-driven progress of malignant diseases [25], [26].

Experimental studies in mice showed that CD4+CD25+Treg do not only play a central role in the maintenance of immunological tolerance but that Treg are also potent inhibitors of antitumor immune responses [27], [28]. Previous studies have examined the suppressive capacities of CD8+CD25+Foxp3+ T cells in colorectal cancer tissues [29]. In CRC specimens the absolute number of CD8+CD25+Foxp3+ T cells were low (<5%). In addition, these cells were absent in most normal tissue samples but present in most CRC specimens; however their clinical relevance remains unknown [29]. Several groups observed Foxp3 expression by various cancer types and its potential role on immune surveillance by producing immunosuppressive cytokines such as IL-10 and TGF-β [30].

Finally, we analyzed the overall survival of patients with low or high Foxp3+ Treg infiltration in the tumor in comparison to the survival of patients with low or high Foxp3 expression of their cancer cells. Those patients with high Foxp3+ expression profile in cancer cells were associated with a poorer prognosis than patients with low expression profile of Foxp3+ in their cancer cells. This correlation of high Foxp3 expression pattern with poor prognosis was not observed for infiltrating Treg in the tumor. Cancer cells expression of Foxp3 followed by secretion of immunosuppressive cytokines into the microenvironment of the tumor tissue may give the tumor a powerful tool to circumvent immunological destruction. Interestingly, an inverse correlation between the number of Foxp3+ Treg and the level of Foxp3+ cancer cells was observed in our patients with CRC suggesting an anti-proliferative effect of TGF-β on Treg.

In conclusion, despite the fact that the sample size in this cohort is small to draw definitive conclusions, our study shows for this first time that high Foxp3 expression in cancer cells is associated with a dismal prognosis in CRC. Multivariate Cox regression analysis demonstrated lymph node metastasis (N category) and Foxp3 expression in colon cancer cells as significant prognostic parameters of survival in human CRC.

## Materials and Methods

### Ethics statement

Ethical approval for this research was obtained from the Human Research Ethics Committee of the University of Wuerzburg. All patients providing tumor tissue as well as normal colon tissue samples signed a consent form prior to surgical removal of the intestinal cancer to allow for this research to be undertaken.

### Patients and controls

Sixty-five patients with histologically confirmed CRC cancer undergoing curative surgical resection in our department between 01/2001 and 06/2004 were included in the study. The histological stage of the tumor was determined according to the Union Internationale Contre le Cancer (UICC)-TNM staging system [31]. Tumors were evaluated for location, stage, and differentiation grade. Data concerning age, gender, level of wall infiltration, and lymph node metastasis were collected in a database. Regular medical visits of the patients after curative therapy were performed at intervals according to the governmental guidelines for tumor patients. Patients, who underwent any neoadjuvant treatment or R1 resection were excluded from analysis. Tumor tissue samples as well as normal colon tissue samples from the patients were frozen instantly in liquid nitrogen, and stored at −80°C until analyzed. Normal colon tissues from healthy individuals served as controls (n = 10). Immunohistochemical analysis confirmed that no analyzed tumor from the patient cohort expressed hMLH1 and hMSH2 indicative for microsatellite instability (positive mismatch repair [MMR] status). The mucinous phenotype of CRC could be associated with false positive subcellular reactions by immunohistochemistry and was therefore excluded from our study. Clinical characteristics of the study population are summarized in ***Table 3***. All patients completed at least a 60 months follow-up after resection.

### Cell culture

The human colon cancer cell lines SW620, SW480, and HCT-116 were obtained from ATCC (Manassas, VA) and cells were cultured at 37°C in 5% CO_2_ using RPMI 1640 Medium (Life Technologies, GIBCO, Carlsbad, CA) supplemented with 10% (v/v) fetal bovine serum (Life Technologies, GIBCO, Carlsbad, CA), 1% (v/v) L-glutamine (Biochrom AG, Berlin, Germany) and 1% (v/v) penicillin/streptomycin (Biochrom AG, Berlin, Germany).

### Flow cytometry analysis (FACS)

Cancer cells from the human colon cancer cell lines SW620, SW480, and HCT-116 were harvested at an exponential growth phase using enzyme free cell dissociation solution (Merck Millipore, Billerica, MA) and analyzed for Foxp3 expression. After washing with dPBS (Life Technologies, GIBCO, Carlsbad, CA) twice, 5×10^5^ cells were treated with Foxp3 staining buffer kit (Miltenyi Biotec, Bergisch Gladbach, Germany) according to the manufacturer's instructions and incubated with PE-conjugated antibody Foxp3 or IgG1 (Miltenyi Biotec, Bergisch Gladbach, Germany) for 30 min at 4°C in the dark. The fluorescence of Foxp3 was measured and analyzed with a FACS flow cytometer (Coulter EPICS XL, Beckman Coulter, Brea, USA).

### Immunohistochemical and immunofluorescent staining

CD4 and CD25 antibodies were provided by Dako (Hamburg, Germany). Two different Foxp3 antibody clones (ab22510 mouse monoclonal and ab2481 goat polyclonal) were purchased from Abcam (Cambridge, UK), TGF-β antibody was provided by Serotec (Duesseldorf, Germany), and IL-10 antibody by R&D Systems (Wiesbaden-Nordenstadt, Germany). Isotype control antibodies were purchased from eBioscience (San Diego, USA). Secondary antibodies used for immunofluorescence double staining and immunohistochemistry were provided by Jackson ImmunoResearch Laboratories Inc. (Suffolk, England). Secondary EPCAM and CD4 antibodies were FITC-conjugated AffiniPure Donkey anti-goat IgG and secondary antibody of Foxp3, IL-10, and TGF-β was Cy3-conjugated AffiniPure Donkey anti-mouse IgG at a 1∶200 dilution (Jackson ImmunoResearch).

For cytospin preparations colorectal carcinoma cells from the patients with CRC were cultured in short-term primary cultures and established human colon cancer cell lines were harvested at an exponential growth phase using enzyme free cell dissociation solution (Merck Millipore, Billerica, MA). After washing with dPBS (Life Technologies, GIBCO, Carlsbad, CA) twice, cells were adjusted to a concentration of 2×10^5^ cells/ml. Cytospins were performed with 50 µl cell suspension at 550 rpm for 1 min in a Cytospin4 Cytocentrifuge (Thermo Fisher Scientific, Waltham, MA).

The staining of CD4, CD25, Foxp3, TGF-β, and IL-10 was performed on serial cryostat sections of the 65 snap-frozen CRC specimens in early-stage tumors (UICC I/II) and late-stage tumors (UICC III/IV) with neighbouring normal colon tissue and 10 normal colon specimens. All tumors stained positive for cytokeratin-20 (CK-20) (Dako, Hamburg, Germany) and negative for cytokeratin-7 (CK-7) (Dako), a pattern characteristic for colonic adenocarcinoma [32]. First we assessed H.E. sections from each tumor tissue to differentiate between cancer cell areas, stromal areas and infiltrating immune cells. We then stained for CD4, CD25, Foxp3, TGF-β, and IL-10 in following serial sections. For Foxp3 expression cytoplasmic, perinuclear cytoplasmic, and nuclear staining pattern were differentially determined [30]. Serial cryostat sections (5 μm) were incubated with the primary antibody or control antibody and with secondary FITC-conjugated (Fluoresceinisothiocyanate) antibody. Then the slides were subsequently incubated with the second primary antibody followed by incubation with secondary Cy3-conjugated antibody. Slides were counterstained with DAPI (4′,6-Diamidino-2-phenylindoldihydrochlorid) (Sigma-Aldrich, Steinheim, Germany) and covered with Polyvinyl-alcohol mounting medium (DABCO, Sigma-Aldrich) and analyzed using a Zeiss camera (Oberkochen, Germany).

For immunohistochemistry, the pre-treatment (fixation) of the slides was the same as described for immunofluorescence. After incubation with the primary antibody, we used a horseradish peroxidase (HRP)-conjugated AffiniPure Donkey anti-mouse or a Donkey anti-goat IgG (Jackson ImmunoResearch). Slides were subsequently incubated in DAB (3,3′-diaminobenzidine) (Biogenex, San Ramon, USA), counterstained with Haemalaun and mounted with Glycergel (Dako, Hamburg, Germany). The quantification of each immunoenzymatic staining of tumor cells out of 65 individual tumor tissues in six individual magnified fields (×400 magnification) for each staining sample was scored by cell counting performed by two independent investigators blinded for the underlying disease. The magnified fields were representative for the whole tumor section. The result of the staining was expressed in percentages (%) positivity. All values were expressed as mean ± SD.

### Real-time quantitative reverse transcription-PCR analysis

To analyze gene expression of CD4, CD25, Foxp3, TGF-β, and IL-10 by RT-qPCR, we extracted total cellular RNA using the RNeasy Minikit from Qiagen (Hilden, Germany). Areas of interest (only epithelial regions) for each tissue section were manually microdissected using a scalpel blade. Equal amounts of tissue areas were assessed (2×1.5 cm^2^ surface area per section, thickness of 10 μm). RNA extraction of patient samples and established human colon cell lines (for Foxp3) was performed according to the manufacturer's instructions. Primer sets were obtained from Qiagen, 18S RNA primer pairs (forward: TCA AGA ACG AAA GTC GGA GGT TCG, reverse: TTA TTG CTC AAT CTC GGG TGG CTG) were designed by Biomers (Ulm, Germany). Matched human colon cDNA was purchased from Pharmingen (Heidelberg, Germany) as control and was standardized to baseline. The housekeeping genes Glyceraldehyde-3-phosphate dehydrogenase (GAPDH), ß-actin, and 18S RNA [33] were used for relative quantification and cDNA quality control. All PCR reactions were carried out with a DNA Engine Opticon 2 System (MJ Research, Biozym, Oldendorf, Germany). The relative quantification value, fold difference, was expressed as 2^−ΔΔCt^. For the analysis in colon cancer cell lines expression is indicated in mean value, ΔCt and relative expression (Foxp3/Housekeeping genes).

### Statistical analysis

Statistical analysis was performed using SAS 9.2. Overall survival was defined as the time period between randomisation and death of any cause. Patients, who were lost to follow-up were censored at the date of last contact. The overall survival was evaluated by means of PROC PHREG (Cox Proportional Hazards Model). The parameters of prognostic potential, identified in a stepwise procedure, have been further investigated by Kaplan-Meier method (PROC LIFETEST). For univariate analysis mean cut-off value for either high or low expression was set at 12% for Foxp3 in tumor infiltrating Treg and 16% for Foxp3 in cancer cells. Univariate analysis of significance for Foxp3 expression of tumor infiltrating Treg and cancer cell expression differences in survival curves were evaluated by Log-rank test. In the same way survival curves were compared for N and T categories as well as primary tumor.

Two independent groups of patients were analyzed using Student's t test (Satterthwaite). More than two groups were analyzed applying PROC GLM (analysis of variances) with post-hoc testing (Tukey). Frequency distributions were compared using kxm tables (Chi-quadrat). Pearson's correlation coefficient was used to describe and to test bivariate correlations. A p-value of less than 0.05 was considered statistically significant.
